# Social Capital as a Mediator in the Link between Women’s Participation in Team Sports and Health-Related Outcomes

**DOI:** 10.3390/ijerph18179331

**Published:** 2021-09-03

**Authors:** Yuval Paldi, Daniel S. Moran, Orna Baron-Epel, Shiran Bord, Elisheva Benartzi, Riki Tesler

**Affiliations:** 1 Department of Health Systems Management, Faculty of Health Science, Ariel University, Ariel 40700, Israel; danielm@ariel.ac.il (D.S.M.); riki.tesler@gmail.com (R.T.); 2 Faculty of Social Welfare and Health Sciences, School of Public Health, University of Haifa, Haifa 31905, Israel; ornaepel@research.haifa.ac.il; 3 Department of Health Systems Management, The Max Stern Yezreel Valley College, Yezreel Valley 1930600, Israel; shiranb@yvc.ac.il; 4 College of Law and Business, Bar Ilan University, Ramat Gan 5290002, Israel; elibenartzi@gmail.com

**Keywords:** team sports, women, social capital, parallel mediation model, self-reported health, psychosomatic symptoms, depressive symptoms

## Abstract

The role of social capital in the association between team sports and health-related outcomes has not been well established in the literature. The purpose of this study was to explore whether social capital components (social support, trust, and social involvement) mediate the association between team sports and health-related outcomes (self-reported health, psychosomatic symptoms, and depressive symptoms). In a cross-sectional research design, we obtained data from 759 participants in the Mamanet Cachibol League, a community team sports model for women in Israel, as well as a comparison group of 308 women who did not participate in any team sports. Team captains were sent a link with an online questionnaire, which were then delivered to team members via text message. Using three parallel mediation models, we found that social support mediated the association between team sports and self-reported health, psychosomatic symptoms, and depressive symptoms. Trust mediated the association between team sports and both psychosomatic symptoms and depressive symptoms. Social involvement was not found to be a mediator in the association between team sports and any of the health-related outcomes. Our findings reveal the important role of social capital, specifically social support and trust, in promoting the health of women who participate in team sports.

## 1. Introduction

A considerable body of literature suggests that an association exists between social capital and various health-related and wellbeing outcomes [1,2,3,4]. Individuals with high levels of social capital report better physical and mental health [5,6], as well as overall happiness and life satisfaction [7,8]. Many studies have linked participation in team sports with increased social capital, suggesting that team sports can serve as a social platform for individuals where it is possible to make connections and exchange resources [9,10,11,12]. While social capital has been identified as a mediating factor between intervention programs and desired outcomes in public health [13], few studies have examined the role of social capital in the association between team sports and health-related outcomes. The current study examined the association between health-related outcomes and participation in the Mamanet Cachibol League (MCL), including the role of social capital as a mediating factor. MCL is a community team sports model that incorporates team sport activity within a social framework for women from different population groups in Israel.

### 1.1. Social Capital

Social capital has been defined by Coleman [14] as “not a single but a variety of different entities having two characteristics in common: They all consist of some aspect of social structure, and they facilitate certain actions of individuals who are within the structure”. According to Bourdieu [15], “Social capital is the sum of the resources, actual or virtual, accumulated by an individual or a group due to their network of different types of relationships based on mutual acquaintance and recognition”. Putnam definition of social capital was “features of social organization such as networks, norms, and social trust that facilitate coordination and cooperation for mutual benefit” [16]. In the current study, we adopted Putnam’s definition, as it emphasizes participation of individuals in structured organizations, such as community sports clubs.

Social capital has also been identified in the literature as consisting of two distinct aspects: (1) A structural aspect, which refers to involvement in social organizations or social networks (including civic involvement) and (2) a cognitive aspect, which refers to an individual’s perceptions of the level of interpersonal trust, sharing, and reciprocity. Structural social capital can be readily observed from the existence of network ties, while cognitive social capital is intangible, as it relates to an individual’s beliefs and feelings [1].

Another distinction regarding social capital refers to individual and community levels. At the individual level, social capital is perceived as directly related to individuals and their relationships. Community-level social capital is defined as the overall achievement of a broad group of people belonging to a given social group. Previous literature has suggested that an individual’s level of social capital has been consistently related to positive health-related and wellbeing outcomes [17,18,19]. Therefore, the current study examines cognitive social capital at the individual level.

### 1.2. Social Capital and Health Outcomes

The concept of social capital plays a key role in public health. Many studies have indicated strong and consistent associations between social capital, particularly cognitive social capital at the individual level, and health-related outcomes, such as depression and other mental health conditions, hospitalization, and mortality [1,3,20].

Social capital is comprised of several key components including social support (by friends and family members); trust (in other people and institutions); and social involvement (volunteering and participation in social activities). Each of these components has been found to be associated with health-related and wellbeing outcomes. Social support from relatives, friends, and neighbors has been found to be negatively associated with depression [21,22] and psychosomatic symptoms [23], while positively associated with self-rated health [24]. Social trust has been found to be negatively associated with depression, depressive symptoms [22,25,26,27], and psychosomatic symptoms [23,28]. Trust has also been found to be positively associated with self-rated health [28,29,30], subjective wellbeing, and life satisfaction [29,31,32]. Volunteering and social involvement have also been found to be positively associated with self-rated health [24,33], subjective wellbeing, and happiness [33,34].

The role of social capital varies in the framework of public health interventions. In some cases, social capital serves as a mediating factor between an intervention and the desired health outcomes [13]. For example, a change in community-related health outcomes following an intervention might be mediated by gains in social capital. This type of mediating effect leveraging social capital as an intervening factor is evident in interventions related to improving urban green spaces [13]. Social capital has also been found to be a mediating factor in the relationship between education and depression among older adults in China, with higher social capital related to a lower risk of depression [35]. Regarding participation in sports, a study that focused on the effects of sports participation on the rate of depression and suicide ideation among adolescents found social support to be a mediating factor that further attenuated the associations with these two risk factors [36].

### 1.3. Social Capital and Sports

Many studies have found an association between sports and social capital. Sports are viewed as a social platform where individuals can meet each other, enjoy being together, and often serve as a base for social networks [16,37]. Participation in sports contributes to the development of pro-social behavior where individuals develop social connections, trust, and active participation, all of which provide a foundation for building local communities [38,39,40,41,42].

Team sports provide an organizational framework that promote active and healthy lifestyles, as well as a social network that can play a major role in promoting motivation and perseverance [43,44]. Team sports have a unique social dynamic, expressed in group cohesion, mutual support, and sense of belonging among its members [45,46]. Studies have shown that any specific group serves as a rallying, supportive, and strengthening body, which increases a sense of belonging, motivation, and persistence; activity in the group enables participants to experience social interactions along with shared social values [43,47]. 

It has been shown that sports, and in particular team sports, are connected to social capital measures including social network expansion, social trust, social engagement, social support, and social involvement. Painter and Price [11] found that participants in recreational soccer leagues made new friends and gained employment opportunities. Harvey et al. [48] showed that team sports in community organizations were related to measures of an individual’s social network. Brown, Hoye, and Nicholson [49] found that members of community sports organizations, when compared to members of other community organizations, had higher levels of trust and better relationships with neighbors. Ball et al. [50] found that participation in sports is positively correlated with mutual trust and social activity. Schüttoff et al. [51] claimed that sports foster greater social involvement. Pawlowski et al. [52] reported that participation in sports groups leads to social support. Skrok et al. [10] argued that sports can positively influence the size and depth of social networks and foster pro-social behavior, while Biernat et al. [53] suggested that sports might improve social involvement. 

A longitudinal study on the personal contribution of participation in the MCL found an increase in social capital among women who participated in the MCL, although they had higher levels of social capital when they joined the league, when compared to non-participants [54]. An additional cross-sectional study on the MCL indicated that participation in the MCL was associated with higher levels of social capital compared to the control group, suggesting a difference in social capital between participants and non-participants [55].

**Figure 1:**
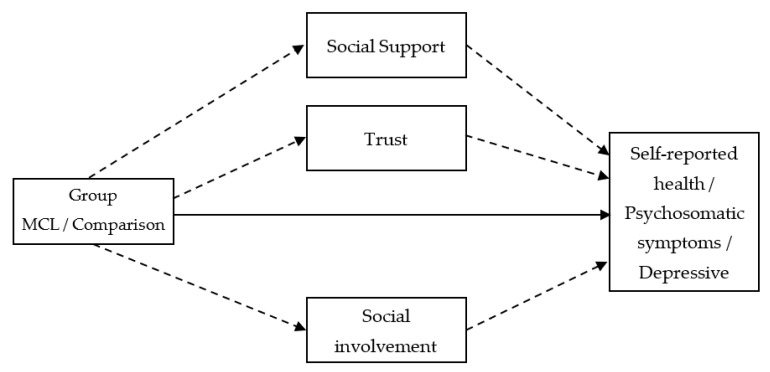
The parallel mediation models tested direct and indirect effects. Note: Group-Comparison (0)/MCL (1); Full line-direct effect; Dashed line-indirect effect.

The purpose of this study was to explore whether social capital components (social support, trust, and social involvement) mediated the association between team sports and health-related outcomes (self-reported health, psychosomatic symptoms, and depressive symptoms). Specifically, we hypothesized that (a) participation in the MCL would be positively associated with self-rated health (SRH); (b) participation in the MCL would be negatively associated with psychosomatic symptoms; (c) participation in the MCL would be negatively associated with depressive symptoms; (d) social support, trust, and social involvement would mediate the relationship between participation in the MCL and self-reported health; (e) social support, trust, and social involvement would mediate the relationship between participation in the MCL and psychosomatic symptoms; and (f) social support, trust, and social involvement would mediate the relationship between participation in the MCL and depressive symptoms. Hypotheses a–c reflect the direct effect and hypotheses d–f reflect the indirect effect of team sports on health-related outcomes (Figure 1).

## 2. Materials and Methods

### 2.1. Participants

The sample included 1067 women (who were mothers) who were recruited in person from different cities located in central Israel. There were 759 participants who were part of the Israeli MCL for at least one year. The comparison group included 308 women who did not participate in the MCL or any other team sports. Inclusion criteria for both groups included women aged 22–55 years (the ages of the youngest and oldest participants) who had at least one school-aged child.

### 2.2. Procedure

MCL participants were recruited with the help of the MCL organization, which provided the contact details of the captains of the teams. We sent the captains a link for an online questionnaire, and in turn, they delivered it to their team members via the WhatsApp application (text message). Comparison group participants were recruited from a large Israeli Internet panel, which includes 100,000 people. An online questionnaire was sent to potential participants via the panel’s on-line system. The study was approved by the institutional ethics committee of Ariel University. All participants were voluntarily recruited and informed of the objectives of the study. Participants also completed an informed consent form and were assured that they were free to withdraw from the study at any point, that their responses would remain confidential, and that the questionnaires would be analyzed anonymously.

### 2.3. Sampling Method

We sampled MCL teams from different geographic regions and socio-economic levels (low, medium, high) according to the Israeli Central Bureau of Statistics [56]. All team members of MCL practice organized team sports approximately twice a week. Participants for the comparison group were randomly sampled from the Internet panel. We included mothers who did not participate in any organized team sports and who had similar demographic, socio-economic, and geographic characteristics of those in the study group (MCL group).

Comparison between the respondents revealed no significant differences between the groups for these variables. Table 1 presents demographic characteristics for the MCL and comparison groups.

### 2.4. Instruments and Measures

The Social Capital Scale [57] refers to various aspects of social capital: Social support, trust, and social involvement. Social support refers to perceptions of general social support from others and includes five items (e.g., “How many close friends do you have?”). Trust refers to one’s perceptions regarding the degree to which others can be trusted and includes three items (e.g., “In your opinion, would most people try to take advantage of you if given a chance, or would they try to be fair to you?”). Social involvement refers to one’s perception regarding the degree to which one is socially involved, and includes four items (e.g., “To what extent have you participated in any community event in the past six months?”). Scale scores range between 1 and 5, with higher scores representing greater social capital. 

To validate the three-dimension construct of the Social Capital Scale in the present sample, a principal components factor analysis with oblique rotation was conducted on its 12 items. Since rotated factors were only moderately correlated (r = 0.27), the data were reanalyzed adopting a Varimax factor rotation [58]. The number of factors to retain was determined by parallel analysis, a method that compares the observed factor strengths with simulated strengths under a noise-only model. There is a growing consensus that this stopping rule is an optimal solution to identify the correct number of components and provides more accurate estimates of the number of factors to retain than Kaiser’s criterion of eigenvalues > 1 [59,60]. A parallel analysis on the 12 items that was based on the mean eigenvalues and the 95th percentile eigenvalue obtained from random data, using 100,000 iterations, indicated a three-factor solution accounting for 55.9% of the variance. The first three observed eigenvalues were 3.91, 1.56, and 1.24 compared with 1.17, 1.26, and 1.09 for the 95th percentiles, respectively, of the randomly generated data. The fourth eigenvalue was 0.78. The first factor (33.23% explained variance) included the five social support items (loadings ranging between 0.52–0.73), the second factor (12.97% explained variance) included the three trust items (loadings ranging between 0.81–0.82), and the third factor (10.37% explained variance) included the four social involvement items (loadings ranging between 0.54–0.78). Reliabilities of the three factors were adequate as indicated by the satisfactory levels of internal consistency: Cronbach’s alphas were 0.74, 0.79, and 0.66, for the social support, trust, and social involvement factors, respectively. Based on these results, three factor scores were computed by averaging the item ratings on the relevant factors, with higher scores indicating higher levels of social support, trust, or social involvement. 

The SRH scale was considered to reflect the physical and functional aspects of health [61]. It was measured using the standard question: “Generally, how do you evaluate your health?” which participants rated using a 6-point scale ranging from 1 (“very bad”) to 6 (“excellent”) [62]. 

Psychosomatic symptoms included questions regarding stomachaches, headaches, backaches, irritability or bad temper, nervousness, and dizziness [63]. Participants rated how often they experienced each symptom during the prior six months to the study on a 5-point scale ranging from 1 (“rarely or never”) to 5 (“about everyday”). In the present study, the scale showed adequate reliability: α = 0.72. An overall psychosomatic symptom score was computed by averaging the ratings on the six items, with higher scores representing a greater extent of symptoms experienced.

The Center for Epidemiological Studies Depression Scale [64] was used to test depressive symptoms; the questionnaire rates the prevalence of eight negative and positive emotional states. Participants rated how frequently during the prior week they felt depressed, sad, lonely, happy, enjoyed of life, felt initiative or lack thereof, felt that everything they did demanded effort, and experienced restless sleep. The scale ranged from 1 (“never or almost never”) to 4 (“all or almost all the time”). The internal consistency was α = 0.76. Based on the results, an overall depression score was computed by averaging the ratings for all the items, with higher scores representing more depressive symptoms.

### 2.5. Data Analysis

Data screening revealed that there were no missing data. Descriptive statistics and Pearson correlations were calculated for the study variables, and group differences across the study variables were tested. The PROCESS macro (Ver. 3.5) for SPSS (Model 4) [65] was applied to examine the mediating effects of social capital dimensions in the link between participation in the MCL and health-related outcomes. Testing the significance of the indirect effects was conducted using bootstrap confidence intervals (CIs) based on 10,000 random samples [65]. An effect is regarded significant if the CIs do not include zero. Since the predicting variable (i.e., group) was dichotomous, unstandardized coefficients for all the effects were calculated. Family-wise significance level for all analyses was 0.05.

## 3. Results

### 3.1. Preliminary Analyses

Descriptive statistics and intercorrelations for all the study variables are presented in Table 2. All variables were significantly correlated in the predicted directions. The three social capital components were positively intercorrelated and negatively associated with the three wellbeing variables, which were also all positively intercorrelated. Moreover, the intercorrelations between the social capital variables and wellbeing variables were moderate, thereby justifying their inclusion in the models. These bivariate associations provide evidence to support the test of mediation analyses.

A one-way MANOVA on all the social capital and health-related variables with study group (MCL, comparison) as independent variables proved to be highly significant (F [6, 1060] = 40.28, Wilk’s Lambda = 0.81, *p* < 0.001). The MCL group showed higher levels of social capital and better wellbeing as compared to the comparison group (Table 3).

### 3.2. Parallel Mediation Models 

**Figure 2:**
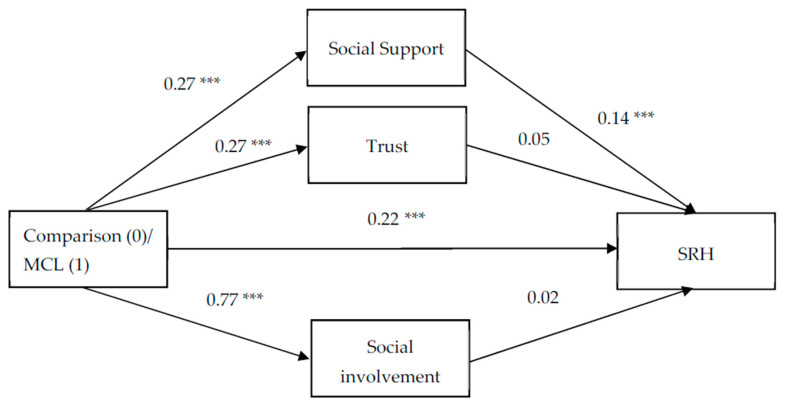
The mediation effect of social capital components on the link between participation in the Mamanet Cachibol League and self-reported health (SRH) (unstandardized coefficients). *** *p* < 0.001.

**Figure 3:**
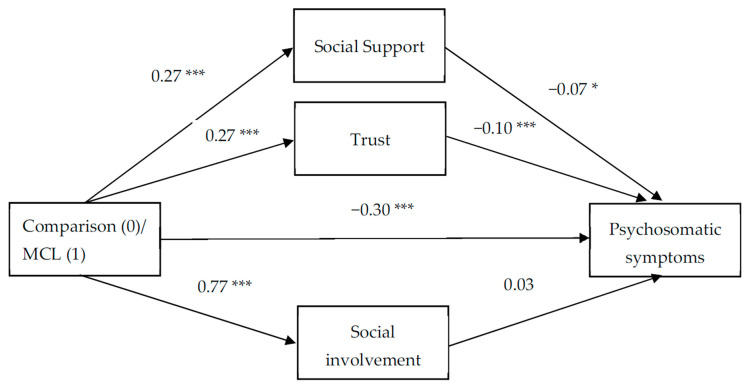
The mediation effect of social capital components on the link between participation in the Mamanet Cachibol League and psychosomatic symptoms (unstandardized coefficients). * *p* < 0.05; *** *p* < 0.001.

**Figure 4:**
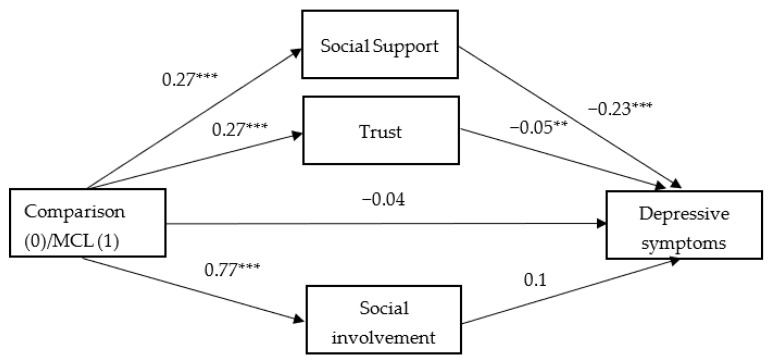
The mediation effect of social capital components on the link between participation in the Mamanet Cachibol League and depressive symptoms (unstandardized coefficients). ** *p* < 0.01; *** *p* < 0.001.

To test the study hypotheses, three parallel mediation models examined the degree to which the three components of social capital mediated the link between participation in the MCL and each of the three health-related outcomes. The mediation model allowed us to explore whether the link between two variables was explained, either fully or partially, by a third mediating variable. A parallel mediation model offered the added benefit of exploring multiple mediators simultaneously, therein providing mediating effect values for each indirect path while accounting for the other indirect paths, and also comparing the differences among the indirect paths. In each model, the independent variable was the group (MCL, comparison); the mediators were social support, trust, and social involvement; and the dependent variable was each of the health variables: SRH, psychosomatic symptoms, and depressive symptoms. To test the significance of the indirect effects of participation in the MCL on wellbeing via social capital, we followed the guidelines proposed by Hayes [65] and created 10,000 bootstrapping samples from the original dataset (N = 1067). Across all 10,000 samples, 95% CIs were computed for the upper and lower potential limits of these indirect effects. When zero is not in the 95% CI, one can conclude that the indirect effect is significantly different from zero at *p* < 0.05. Table 4 presents the results of the mediation analyses and Figure 2, Figure 3 and Figure 4 show the coefficients for each parallel mediation model with SRH (Figure 2), psychosomatic symptoms (Figure 3), and depressive symptoms (Figure 4) as dependent variables. 

When treating SRH as a dependent variable (Figure 2), participation in the MCL was shown to be significantly related to each of the mediating variables: Social support (b = 0.27, *p* < 0.001), trust (b = 0.27, *p* < 0.001), and social involvement (b = 0.77, *p* < 0.001), indicating stronger effects of participation in the MCL on social involvement than on social support and trust. However, only social support showed a significant association with SRH (b = 0.14, *p* < 0.001). More importantly, only social support was found to be a significant mediator in the link between participation in the MCL and SRH (b = 0.04, 95% CI 0.01, 0.07). The model coefficients (Figure 2) indicated that participation in the MCL was associated with higher levels of social support, which in turn was related to better SRH. After including the three mediators in the model, the link between participation in the MCL and SRH was still significant (b = 0.22, *p* < 0.001), indicating partial mediation by social support.

Regarding psychosomatic symptoms as a dependent variable (Figure 3), a slightly different pattern emerged. Both social support and trust were significantly associated with psychosomatic symptoms (b = −0.07, *p* = 0.03, b = −0.10, *p* < 0.001, for social support and trust, respectively). More importantly, both social support and trust were found to be significant mediators in the link between participation in the MCL and psychosomatic symptoms (b = −0.02, 95% CI −0.04, −0.001, and b = −0.03, 95% CI −0.05, −0.01, for social support and trust, respectively). However, a contrast analysis revealed no significant difference between the indirect paths via social support and trust (b = 0.01, 95% CI −0.02, 0.04). The model coefficients (Figure 3) indicated that participation in the MCL was associated with higher levels of social support and trust, which in turn was related to lower levels of psychosomatic symptoms. When we included the three mediators in the model, the direct path between participation in the MCL and psychosomatic symptoms was still significant (b = −0.30 *p* < 0.001), indicating partial mediation by social support and trust.

Finally, with depressive symptoms as the dependent variable (Figure 4), both social support and trust were significantly associated with depressive symptoms (b = −0.23, *p* < 0.001, b = −0.05, *p* < 0.01, for social support and trust, respectively). In addition, both social support and trust were found to be significant mediators in the link between participation in the MCL and depressive symptoms (b = −0.06, 95% CI −0.09, −0.04, and b = −0.01, 95% CI −0.02, −0.003, for social support and trust, respectively). The model coefficients (Figure 4) indicated that participation in the MCL was associated with higher levels of social support and trust, which in turn was related to lower levels of depressive symptoms. Yet, as opposed to the findings that emerged for psychosomatic symptoms, a contrast analysis revealed a significant stronger indirect effect via social support than via trust as a mediator (b = −0.05, 95% CI −0.08, −0.03), indicating that participation in the MCL was associated with lower levels of depressive symptoms, more so due to social support than to feelings of increased trust. Including the three mediators in the model, the link between participation in the MCL and depressive symptoms was not significant (b = −0.04, *p* = 0.16), indicating full mediation by social support and trust.

## 4. Discussion

To test the role of social capital as a mediating factor, three parallel mediation models were conducted, each including a different health-related outcome. Overall, the findings support our hypotheses, revealing the role of social capital as a mediating factor in the link between team sports participation (MCL) and health-related outcomes. Specifically, social support was found to be a mediating factor in the link between MCL participation, SRH, and psychosomatic and depressive symptoms. Trust was found to be a mediating factor in the link between MCL participation and psychosomatic and depressive symptoms. Yet, social involvement was not found to have any mediating role. It seems that the social interaction that occurs in team sports, in addition to the bonds that are often formed between participants, are used as resources in improving participants’ states of mind. These findings contribute to the evidence that the social nature of team sports may play an important role in fostering health-related outcomes among women.

Due to the non-experimental cross-sectional nature of our study, it is not possible to confirm a causal relationship between study variables, or to dispute the assumption that women who participate in team sports have higher levels of social capital to begin with. However, as shown by Baron-Epel et al. [54], although women who participated in the MCL had high levels of social capital prior to joining the league, participation further increased their social capital level. Our study focused on the difference between the three mediators, rather than the cause–effect associations between team sports and social capital. 

We found a direct positive effect of participation in the MCL on SRH, which was one of our hypotheses. Our results are aligned with two other studies that suggested that older women who participated in a walking group had higher SRH statuses compared to women who walked on their own [66,67]. In an additional study, engagement in group sports activities (specifically golf and walking) was significantly related to excellent self-rated health [68]. In a further study, Russell and Chase (2019) found that participation in social activities was inversely associated with sedentary behavior and multiple dimensions of health statuses [69]. We hypothesized that social capital components would mediate the relationship between participation in the MCL and SRH, though found that only social support was a significant mediator. This finding is consistent with previous studies, which have indicated that participation in sports activities positively impacts social relationships. Sport activities can, therefore, be considered as a tool for fostering positive and meaningful relationships between participants [10]. 

We also found a direct negative effect of participation in the MCL on psychosomatic symptoms, another one of our hypotheses. Previous studies have shown that women who participated in team sports had lower psychosomatic symptoms compared with non-participants [54,55]. An additional study that investigated the cross-sectional and longitudinal associations among leisure time physical activity, mental wellbeing, and subjective health in middle-aged adults found associations between participation in group and team sports and psychosomatic symptoms [70]. We also hypothesized that social capital components would mediate the relationship between participation in the MCL and psychosomatic symptoms; our findings showed that only social support and trust were significant mediators (social involvement was not). The role of social support as a mediating factor between MCL participation and psychosomatic symptoms is in line with previous studies that have evaluated group physical activity interventions among young women and girls. Stromback et al. [71] reported that young women who participated in a body-based physiotherapeutic group intervention showed a significant psychosomatic symptom reduction. Duberg et al. [72] reported that teenage girls who participated in a group dance intervention showed a significantly greater reduction in psychosomatic symptoms in comparison with a control group. The authors of both studies concluded that the post-intervention reduction in psychosomatic symptoms, as well as other mental health issues, was possibly due to the increase in social support. The role of trust as a mediating factor between MCL participation and psychosomatic symptoms has not been well established in the literature. Several studies have reported associations between participation in team sports or involvement in community sport organizations and higher trust in others [49,50]. It has also been reported that people with low social trust have more psychosomatic symptoms compared to people with high social trust [73]. The current study suggests that trust that was formed during team sport participation acts as a resource in enhancing positive feelings, which in turn is related to lower levels of psychosomatic symptoms.

We hypothesized that there would be a direct negative effect of participation in the MCL on depressive symptoms, though no direct effect was found. This finding is in line with Doré et al. [74], who found that team sport and informal group participation in physical activity was not significantly associated with depressive symptoms. In contrast, a massive body of literature suggests negative associations between participation in team or group sports and depressive symptoms [68,75,76].

We also hypothesized that social capital components would mediate the relationship between participation in the MCL and depressive symptoms; results showed that only social support and trust were significant mediators (social involvement was not). Social support was found to be a stronger mediator than trust. The role of social support as a mediating factor in the link between sports participation and depressive symptoms has been reported in the literature among both adolescents [36,77] and adults [78]. Team sports provide positive social support, foster positive interpersonal relationships, and promote teamwork. Two major benefits of team participation include being a part of shared goals and feeling a strong sense of connectedness with other participants. Both of these benefits have the ability to positively influence individuals’ mood [79,80]. In contrast to social support, the role of trust as a mediating factor between MCL participation and depressive symptoms is less established in the literature. Several studies have reported an association between participation in team sports and involvement in community sport organizations with higher trust in others [49,50]. Other studies have shown that low levels of interpersonal trust predict depression symptoms in the long term [81,82]. The current study emphasizes the role of trust, which was formed during participation in team sports as a way to cope with depressive symptoms.

Different health outcomes are potentially influenced by social support in two different ways. The first way relates to different behavioral processes, which include health behaviors. Accordingly, social support can be viewed as health promoting, as it enables healthy behaviors (e.g., exercise and healthy eating). The second way health outcomes are possibly influenced via social support includes psychological processes that are connected with emotion and feelings of control [83]. There also exists an association between trust at the individual level and various health outcomes; this link may include pathways whereby trust reduces stress, which in itself is beneficial to one’s health. Trust has also been shown to promote involvement in different social networks, which also improve health and wellbeing [84].

Our study had a few limitations. First, our findings were based on cross-sectional data that were collected at one point in time, and therefore were not able to consider the effect of the MCL over a long period of time. Second, we were unable to infer causality; longitudinal studies and repeated measures on the same variables are needed in future studies. Next, our findings were based on self-reported data, which may include biased answers. Finally, the MCL participants were more socially oriented than the non-participants due to the nature of the sport; likewise, as sport and/or competition is not appealing to all women, only those interested in participating would have joined the MCL.

## 5. Conclusions

Our study sheds light on the role of social capital components in the link between team sports and health-related outcomes. Firstly, our findings reinforce the important role of social support as a mediator and its priority over the two other social capital components tested in the parallel mediation models (trust and social involvement). The MCL may have contributed to both physical and mental health benefits, in part by fostering the development of social networks, reinforcing social ties, and enhancing social support. Evidence suggests that team sport participation is associated with positive psychological and social health outcomes. It has been concluded that the social aspects of team sports mediate the association between participation and better health [85].

Secondly, our study also emphasized the role of trust as a mediator between participation in the MCL and health-related outcomes in two out of the three parallel mediation models tested. MCL may have contributed to the formation of trust between participants, which in turn may have improved health-related outcomes. The influence of trust on health-related outcomes may have occurred via various mechanisms, such as social support, informal social control, and collective action [30].

Thirdly, in the current study, social involvement was not found to be a mediating factor between participation in the MCL and health-related outcomes in any one of the of the three parallel mediation models tested. A possible explanation is that the MCL model emphasizes physical activity, social interactions, and social support over social involvement.

Finally, in two of the three parallel mediation models tested, the mediation effect was partial, meaning that other unknown variables served as mediators between MCL participation and health-related outcomes. These findings are in line with a review by Kanamori, Takamiya, and Inoue [86], which suggested that social factors (e.g., social support, social network, and social capital) are only one component of the mechanism between group sport participation and health outcomes. The other two components suggested by authors were benefits of physical activity (e.g., inducing good adherence and long duration) and psychological factors (e.g., leading to enjoyment, enhanced self-esteem, and decreased stress).

The findings of our study have shown that it is important to encourage group sport activities for women, especially among those with low levels of social support, trust, and social involvement. We believe that future research should focus on whether similar results would be obtained in different communities, and whether social capital and health indices develop over time among team sport participants. It would also be insightful to examine the development of social capital and health indices in additional group frameworks in order to promote social capital and health.

## Figures and Tables

**Table 1:** Demographic characteristics of the Mamanet Cachibol League group and the comparison group.

Variable	MCL	Comparison	Statistic (*df*)	*p*-Value, 95% CI
	(*n* = 759)	(*n* = 308)		
	% (*n*) or *M (SD)*	% (*n*) or *M (SD)*		
**Age (in years)**	40.48 (5.46)	40.21 (5.00)	*F* (1,1065) = 0.57	0.45 [−0.15, 0.96]
**Education**				
High school or less	74 (9.7)	27 (8.8%)	χ^2^ (2) = 3.28	0.19
Vocational diploma	135 (17.8%)	42 (13.6%)		
Academic degree	550 (72.5%)	239 (77.6%)		
**Number of children**	2.81 (0.86)	2.87 (0.94)	*F* (1,1065) = 0.92	0.34 [−0.03, 0.004]
**Economic status ^a^**	3.46 (0.99)	3.40 (1.09)	*F* (1,1065) =0.77	0.38 [−0.006, 0.004]
**BMI**	24.96 (4.42)	25.17 (4.85)	*F* (1,1065) =0.45	0.51 [−0.64, 0.16]

^a^ Economic status = 1 (much below average) to 5 (much above average); Abbreviations: MCL, Mamanet Cachibol League; SD, standard deviation, CI, confidence interval; BMI, body mass index.

**Table 2:** Descriptive statistics and zero-order correlations for all study variables.

Measure	M	SD	1	2	3	4	5	6
1. Social support (1–5)	4.16	0.64	1					
2. Trust (1–5)	3.41	0.85	0.40 ***	1				
3. Social involvement (1–5)	3.17	0.93	0.45 ***	0.27 ***	1			
4. Self-reported health (1–6)	5.17	0.75	0.18 ***	0.14 ***	0.15 ***	1		
5. Psychosomatic symptoms (1–5)	2.20	0.62	−0.15 ***	−0.19 ***	−0.11 ***	−0.29 ***	1	
6. Depressive symptoms (1–4)	1.61	0.40	−0.41 ***	−0.25 ***	−0.19 ***	−0.25 ***	0.42 ***	1

Note. *** *p* < 0.001; Abbreviations: SD, standard deviation.

**Table 3:** Study variables for MCL and comparison groups.

Variable	MCL (*n* = 759) *M (SD)*	Comparison (*n* = 308) *M (SD)*	*F*	*p*-Value	*Partial eta* Squared
Social support	4.24 (0.61)	3.97 (0.68)	40.29	<0.001	0.4
Trust	3.49 (0.85)	3.22 (0.86)	22.49	<0.001	0.2
Social involvement	3.39 (0.88)	2.63 (0.84)	173.06	<0.001	0.14
Self-reported health	5.26 (0.70)	4.96 (0.82)	34.92	<0.001	0.3
Psychosomatic symptoms	1.92 (0.58)	2.25 (0.66)	63.98	<0.001	0.6
Depressive symptoms	1.58 (0.37)	1.69 (0.46)	15.77	<0.001	0.2

Abbreviations: MCL, Mamanet Cachibol League.

**Table 4:** Direct and indirect unstandardized effects on health components with social capital variables as mediators.

Mediation Model	Estimate	Bootstrapping 95% CI	*t* (1067)	*p*
**Dependent variable: Self-reported health**				
Total effect	0.30		5.91	<0.001
Direct effect	0.22		4.21	<0.001
Total indirect effects	0.07	[0.03, 0.12]	NA	NA
Indirect via social support	0.04	[0.01, 0.07]	NA	NA
Indirect via trust	0.01	[−0.001, 0.03]	NA	NA
Indirect via social involvement	0.02	[−0.02, 0.06]	NA	NA
**Dependent variable: Psychosomatic symptoms**				
Total effect	−0.33		8.00	< 0.001
Direct effect	−0.30		6.89	< 0.001
Total indirect effects	−0.03	[−0.06, 0.01]	NA	NA
Indirect via social support	−0.02	[−0.04, −0.001]	NA	NA
Indirect via trust	−0.03	[−0.05, −0.01]	NA	NA
Indirect via social involvement	0.02	[−0.02, 0.06]	NA	NA
**Dependent variable: Depressive symptoms**				
Total effect	−0.11		3.97	< 0.01
Direct effect	−0.04		1.41	0.16
Total indirect effects	−0.07	[−0.10, −0.04]	NA	NA
Indirect via social support	−0.06	[−0.09, −0.04]	NA	NA
Indirect via trust	−0.01	[−0.02, −0.003]	NA	NA
Indirect via social involvement	0.01	[−0.01, 0.03]	NA	NA

Abbreviations: CI, confidence interval; NA, not applicable.

## Data Availability

The data that support the findings of this study are available from the corresponding author, upon reasonable request.
